# Impact of Multimorbidity Burden on Mortality Risk among Colon Cancer Survivors

**DOI:** 10.7150/jca.103438

**Published:** 2025-01-01

**Authors:** Su-Hung Wang, Mitsuhiro Koseki, Ming‑Jen Sheu, Huang-Lan Li, Ying-Jia Lin, Ching-Chieh Yang, Chung-Han Ho

**Affiliations:** 1 Division of Gastroenterology and Hepatology, Department of Internal Medicine, Chi Mei Medical Center, Tainan, Taiwan.; 2 Department of Internal Medicine, Chi Mei Medical Center, Tainan, Taiwan.; 3 Cancer Center, Chi Mei Medical Center, Tainan, Taiwan.; 4 Department of Medical Research, Chi Mei Medical Center, Tainan, Taiwan.; 5 Department of Radiation Oncology, Chi Mei Medical Center, Tainan, Taiwan.; 6 Department of Pharmacy, Chia-Nan University of Pharmacy and Science, Tainan, Taiwan.; 7 Department of Information Management, Southern Taiwan University of Science and Technology, Tainan, Taiwan.; 8 Cancer Center, Taipei Municipal Wanfang Hospital, Taipei Medical University, Taipei, Taiwan.

**Keywords:** Multimorbidity, Charlson Comorbidity Index, Taiwanese Colon Cancer Survivors, Mortality

## Abstract

**Purpose:** Multimorbidity among colon cancer survivors reflected the coexistence of multiple chronic conditions. This study aimed to understand the comorbidity risks for long-term colon cancer survivors using a real-world population database.

**Methods:** Taiwan cancer registry from 2016 to 2021 identified patients diagnosed with colon cancer, selecting those who survived beyond five years. Charlson Comorbidity Index (CCI) was used to assess the level of comorbidities, categorizing patients into no (CCI=0), mild (CCI=1-2), and severe (CCI≥3) comorbidity groups, for estimating the impact on survival. Cox regression model was applied to estimate risk factors associated with comorbidities among long-term colon cancer survivors.

**Results:** In this cohort study of 13,209 colon cancer survivors, most had no comorbidity (82.23%), following as mild (10.03%) and severe (7.74%) comorbidity. Our study revealed the significant association between higher CCI scores and increased mortality risk. Compared with patients without comorbidities, mild comorbidities patients exhibited a significantly higher risk of mortality (HR:4.56; 95% CI:3.93-5.28), and those with severe comorbidities had an increased risk (HR:12.67; 95% CI:11.15-14.40) after adjusting potential confounders. Subgroup of sex, age, clinical stage, and treatment types show that colon cancer survivors with mild/severe comorbidities had significant higher mortality risk than those without comorbidities.

**Conclusion:** This study indicated the critical role of comorbidity management may improve the survival outcomes for colon cancer patients, particularly those with high-risk factors and severe comorbidities.

## Introduction

With the aging population and shifting disease patterns, the prevalence of multimorbidity is on the rise, particularly in primary care, posing one of the most significant clinical challenges today^1^, ^2^. A national survey in Japan found that the prevalence of multimorbidity among adults over 18 was 29.9%, and 62.8% in those aged 65 and above^3^. The concern around multimorbidity is fueled by its association with increased health risks, such as higher mortality, decreased quality of life, physical and psychological impairments, and greater healthcare utilization^4^. Patients with multimorbidity often experience a heightened treatment burden, including the use of multiple medications and increased frequency of medical interventions^5^, a factor that further exacerbates health risks^6^. This study focuses on the burgeoning issue of multimorbidity, defined as the coexistence of two or more chronic diseases within an individual.

This is particularly pertinent given that two-thirds of cancer patients are reported to have multimorbidity, with common conditions being hypertension, asthma, and cancer, along with other complications like anxiety, depression, and migraines^7^, ^8^. The study emphasizes the importance of primary care in cancer treatment, highlighting the need for integrated, coordinated care, especially in the context of the rising incidence and mortality rates of colon cancer in Taiwan and globally^9^-^12^. The research also addresses the trend of colon cancer affecting increasingly younger populations and the consequent necessity for comprehensive long-term survivor care, considering the high five-year survival rate post-treatment^13^-^17^. Therefore, this study would like to understand the comorbidity risks for long-term colon cancer survivors. The primary objective of this research is to examine the correlation between the burden of multimorbidity and mortality risk in colon cancer survivors.

## Material and Methods

### Study design, setting, and patients

In this research, colon cancer patient data from the Taiwan Cancer Registry (TCR) and the National Health Insurance Research Database (NHIRD) were utilized. Established in 1979, the TCR database is renowned for its high-quality, accurate diagnostic and treatment records, serving as a pivotal resource for monitoring cancer incidence and mortality trends in Taiwan. The TCR expanded in 2002 to include comprehensive datasets for cancers of the colon, rectum, oral cavity, pharynx (excluding nasopharynx), liver, lung, breast, and cervix. Further enhancement occurred in 2007 with the inclusion of prostate cancer data^18^, ^19^.

Data on comorbidities and medical histories were procured from the NHIRD based on Taiwan's National Health Insurance program, including all inpatient and outpatient health services. To facilitate research, the Health and Welfare Data Science Center (HWDC) in Taiwan amalgamated the population database, interlinking it with various health-related datasets. This integration was meticulously managed to ensure compliance with personal information protection laws^20^.

### Ethical statements

This study was conducted strictly according to the Declaration of Helsinki and approved by the Institutional Review Board (IRB) of the Chi Mei Medical Center (CMMC) (IRB serial no. 11210-013). In this study, all participant data were rendered anonymous to maintain confidentiality. The requirement for patient informed consent was exempted due to the retrospective and observational design of the study, as approved by the IRB of the CMMC. This exemption was granted with the understanding that it would not adversely affect the welfare of the patients involved in the study.

### Study population

The TCR database, spanning from 2016 to 2021, was utilized to identify patients diagnosed with colon cancer, classified under International Classification of Disease for Oncology, 3rd Edition (ICD-O-3) codes, C18-C20. The study included patients initially diagnosed with colon cancer who had a minimum survival duration of five years, specifically those whose fifth-year survival occurred between 2017 and 2019. Exclusion criteria encompassed patients diagnosed before the age of 20, individuals who either experienced or had uncertain recurrences within the five-year survival period, and cases with missing data pertinent to the research objectives.

The Charlson Comorbidity Index (CCI) score was used to identify the multimorbidity burden. The CCI was computed, omitting scores attributed to cancer, based on hospitalizations occurring in the fourth- and sixth-year post-diagnosis^21^, ^22^. Patients were stratified into three disease severity categories based on their CCI scores: 0, 1-2, and ≥3. A spectrum of comorbidities including congestive heart failure, peripheral vascular disease, and others were identified using ICD Clinical Modification codes. These comorbidities were considered for inclusion if recorded during hospitalization or in three or more outpatient visits within preceding the cancer diagnosis.

**Figure 1:**
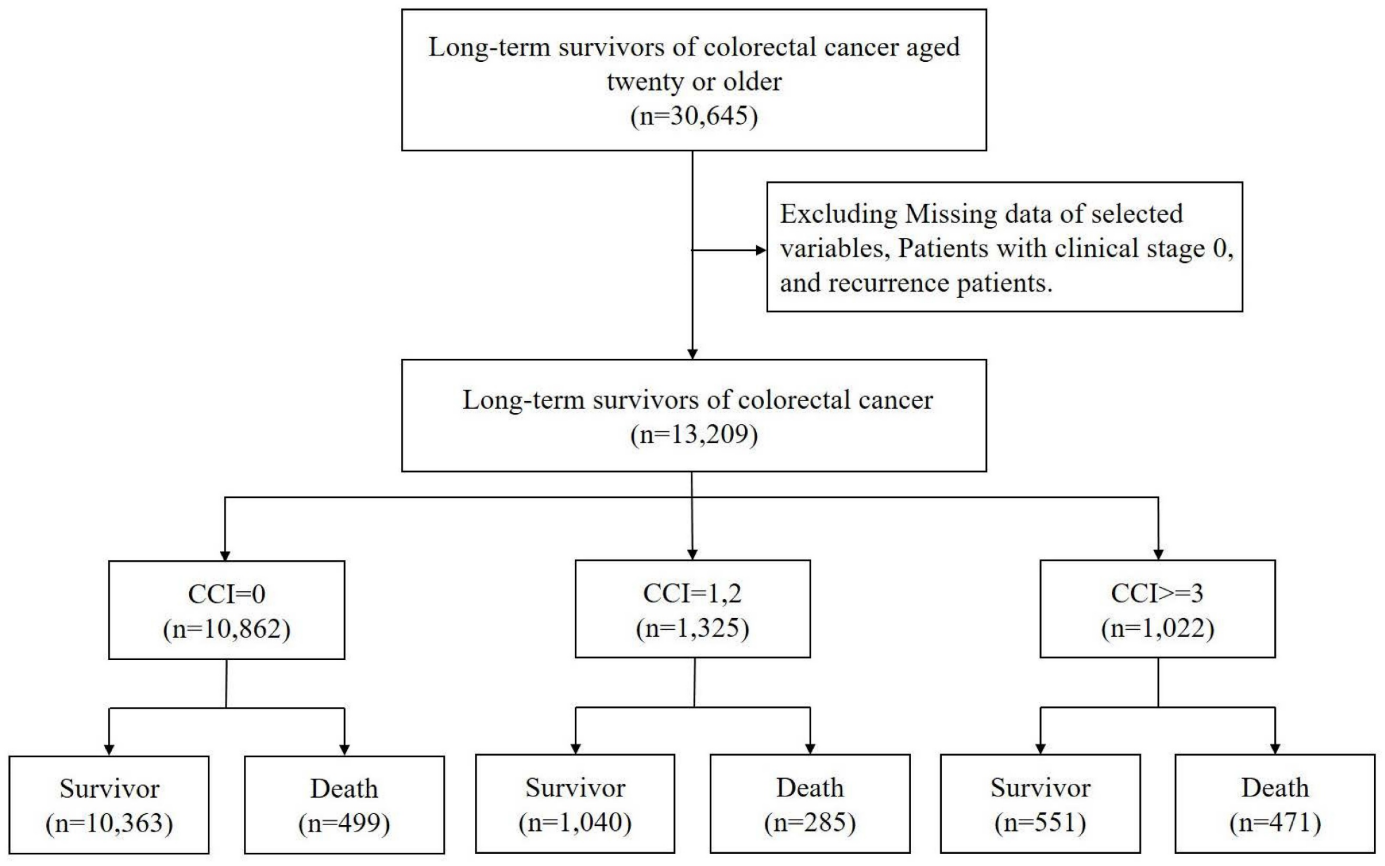
The flow chart of study subjects' selection.

The endpoint of the follow-up period was set at December 31, 2021, marking a three-year follow-up duration. Age analysis was conducted based on the age at the fifth year following the initial diagnosis, which is calculated as the age at diagnosis plus five years (Figure 1).

### Outcome and measurements

The primary objective of this research was to assess the overall mortality rates in long-term colon cancer survivors, categorized based on their CCI scores. A critical aspect of the study was to comprehensively understand the role of comorbidities in affecting survival outcomes and to pinpoint high-risk groups for more focused clinical interventions. Mortality was defined and identified using Taiwan's cause-of-death database. The research methodology entailed an in-depth mortality risk analysis, considering varying comorbidities, and encompassed evaluations based on age, gender, clinical stage, and treatment modalities for each comorbidity category. Baseline characteristics of the patient cohort were meticulously recorded, encompassing demographics (age, sex), body mass index (BMI), lifestyle factors (smoking and drinking habits), and clinical parameters (cancer stage, treatment modalities). Outcomes, such as mortality rates, were meticulously adjusted for confounding variables.

### Statistics analysis

Student's t-test or Wilcoxon rank-sum test was employed to compare the distributional variances of continuous variables between the case and control groups. Concurrently, Pearson's chi-squared test or Fisher's exact test was utilized to assess the distributional discrepancies of categorical variables between these two groups.

To ascertain the risk factors associated with comorbidities among long-term colon cancer survivors, the Cox proportional hazards regression model was applied. Additionally, the Kaplan-Meier plot was used to delineate mortality trends across diverse groups, and the log-rank test was conducted to evaluate differences between these groups. All statistical analyses were performed using SAS 9.4 for Windows (SAS Institute, Inc., Cary, NC, USA). Kaplan-Meier curves were plotted using STATA version 12.0 (Stata Corp., College Station, TX, USA).

## Results

Our study analyzed a cohort of 13,209 colon cancer survivors (Figure.1). Most of these survivors had no comorbidity (CCI=0, n=10862, 82.23%). Those with mild comorbidity accounted for 1325 (10.03%) of the cohort (CCI=1 or 2), while severe comorbidity was observed in 1022 (7.74%) participants (CCI≥3). Mortality rates varied significantly based on the comorbidity status. Of the survivors with no comorbidity, 499 (4.59%) passed away during the follow-up period. The death rate was higher in those with mild comorbidity, with 285 deaths (21.51%) recorded. The highest mortality rate was observed among participants with severe comorbidity, where 471 (46.09%) succumbed.

The mean age of the patients was 68.84 ± 12.65 years, with a difference in age distribution across CCI categories (p < 0.0001) (Table 1). A significant disparity was also observed in the sex distribution, and males presented a higher proportion in higher CCI categories (p < 0.0001). The BMI also indicated a significant difference between these three groups (p < 0.0001). For smoking and drinking habits, patients in severe comorbidity group show significantly higher percentages than other groups. Additionally, patients in severe comorbidity group had significantly more late cancer stage compared with patients without or with mild comorbidity groups (p = 0.0055). The mortality rates exhibited significant differences, with higher rates observed in patients with higher CCI scores (p < 0.0001) for overall mortality and colon-specific death). The mean follow-up time was 1.46 years, showing significant variation across the CCI groups (p < 0.0001).

**Figure 2:**
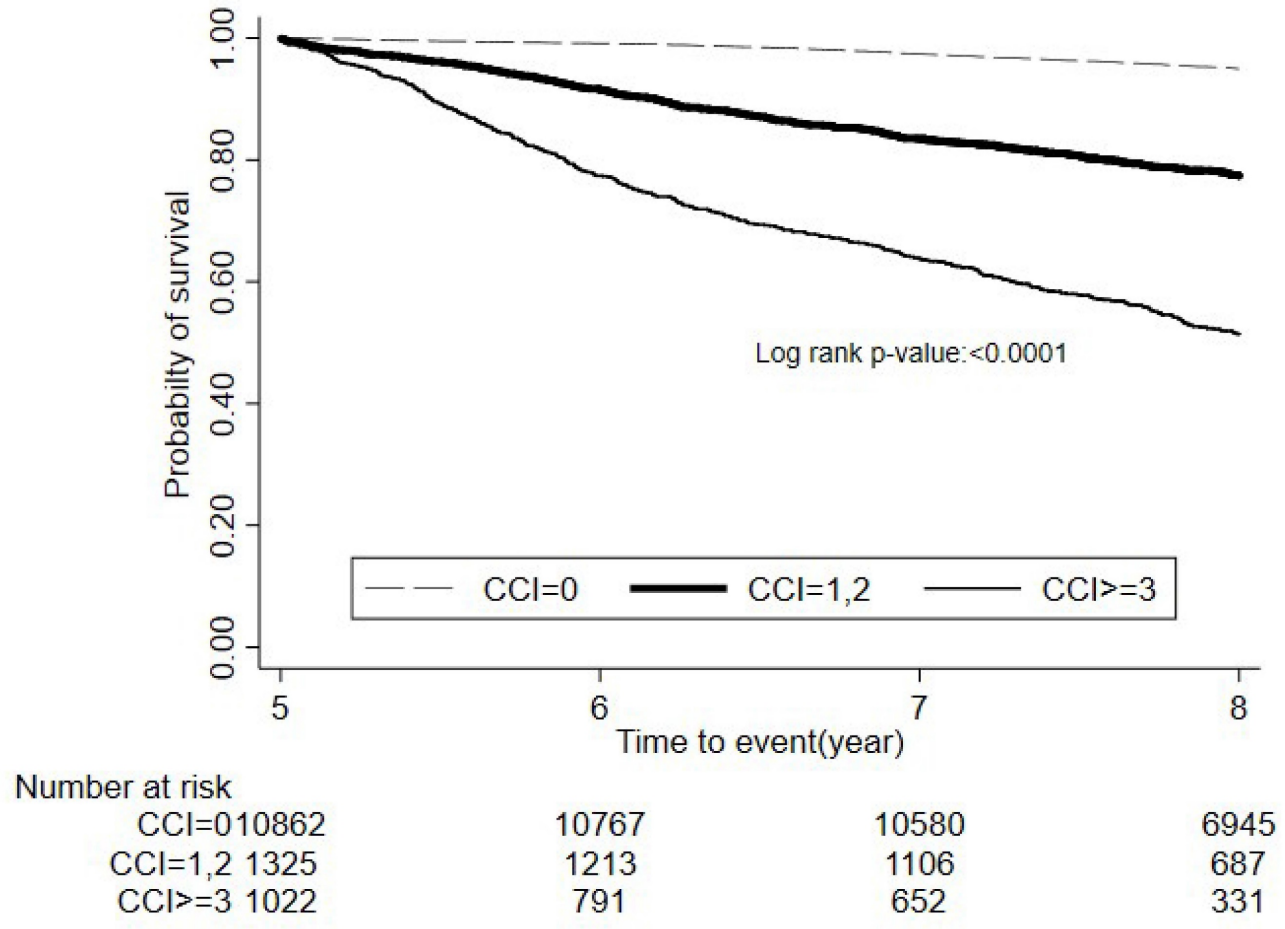
The trend of overall mortality among colon cancer survivors with no, mild, and severe comorbidities. *Adjusted age, sex, BMI, smoking, drinking, cancer stage, surgery, radiotherapy, chemotherapy.

Figure 2 presented Kaplan-Meier survival curves for colon cancer patients stratified by CCI scores over a follow-up study period. The survival probabilities were distinctly lower for patients with higher CCI scores, indicating poorer outcomes with increasing comorbidity. Patients with a CCI score ≥3 exhibited progressively lower survival rates than other two groups with log-rank test: p<.0001.

Table 2 presented the risk of overall mortality among colon cancer patients between three comorbidities burden groups. Compared with patients without comorbidities, patients with mild comorbidities burden group exhibited a significantly higher risk of overall mortality as 4.56(95% CI: 3.93-5.28, p<.0001), and those with severe comorbidities burden increased the risk of 12.67(95% CI: 11.15-14.40, p<.0001) after adjusting for potential confounders listed in Table 1.

**Figure 3:**
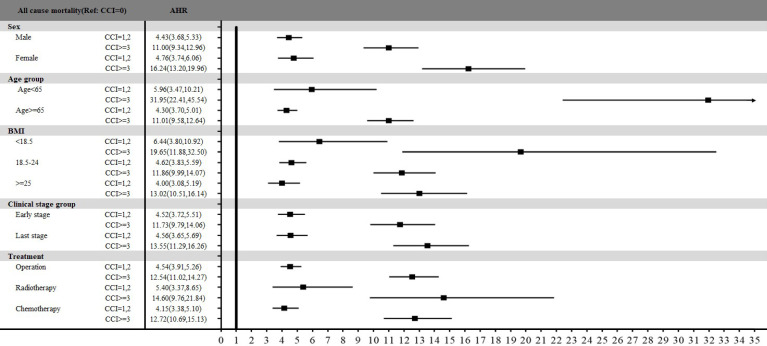
Stratified analysis of all-cause mortality by comorbidity status across sex, age, BMI, clinical stage, and treatment types.

Figure 3 presents the stratified analysis of overall mortality between three groups among sex, age, BMI, clinical stage, and treatment types. All subgroups indicated that colon patients with mild or severe comorbidities had statistically significant higher mortality risk compared with those without comorbidities. Especially, age stratification revealed that patients under 65 years had totally higher AHR of 5.96 (95% CI: 3.47-10.21, p<.0001) for those with mild comorbidities and 31.95 (95% CI: 22.41-45.54, p<.0001) for those with severe comorbidities than those without comorbidities. For patients with different BMI classification, the significant association between comorbidity and mortality risk was also presented among all three BMI groups. Especially, patients with a BMI <18.5 had significantly higher overall mortality compared to patients without comorbidities, with AHR of 6.44 (95% CI: 3.80-10.92, p<.0001) for mild comorbidities and 19.65 (95% CI: 11.88-32.50, p<.0001) for severe comorbidities. Additionally, treatment types also indicated variable impacts on mortality. The AHR for operation, radiotherapy, and chemotherapy at patients with severe comorbidities were 12.54 (95% CI: 11.02-14.27, p<.0001), 14.60 (95% CI: 9.76-21.84, p<.0001) and 12.72 (95% CI: 10.69-15.13, p<.0001), respectively, compared with those without comorbidities.

## Discussion

Of 13,209 colon cancer patients categorized by CCI scores present significant mortality risk among different level of comorbidities groups. This study observed significant differences among groups of colon cancer patients in age, sex, BMI, smoking and drinking habits, cancer stage, and treatment types. The findings of this study indicated that colon cancer patients with mild or severe comorbidities had higher mortality risk than those without comorbidities.

Our study found that older individuals aged over 65 years had higher overall mortality among the three CCI groups. Additionally, some studies revealed that while the stage of colon cancer at diagnosis was key in determining patient outcomes, the presence of comorbidities significantly complicated cancer management and impacted survival^23^-^26^. These findings underscored the requirement for cancer control and treatment research to focus on comorbidity issues relevant to older patients, who often had multiple preexisting conditions, such as hypertension and heart problems, that influenced their mortality risk post-diagnosis.

Our study found that long-term colon cancer survivors in different BMI classification had significantly higher mortality risks of those with mild or severe comorbidities than those without comorbidity, especially for patients with a BMI <18.5. Additionally, it was observed that variations in BMI and lifestyle habits contributed to this trend^27^. A systematic review and meta-analysis encompassing data from 13 studies highlighted that weight gain from early adulthood to midlife is linked to a modest but significant increase in colon cancer risk (HR 1.23, 95% CI 1.14-1.34)^28^. Furthermore, obesity also seems to elevate the likelihood of mortality from colon cancer^29^. Therefore, the nutritional support and careful management of comorbidities should be considered in treatment planning for patients with abnormal BMI levels, as these factors may play a critical role in improving overall survival outcomes.

Cigarette smoking had been identified as a risk factor for various types of colonic polyps, with a notably high risk associated with advanced adenomatous polyps, especially those that are larger and exhibit severe dysplastic features^30^. Additionally, smoking may elevate the risk of colon cancer in patients who have Lynch syndrome, also known as hereditary nonpolyposis colon cancer (HNPCC)^31^. In our study, the prevalence of smoking was also higher. Given these findings, along with numerous other detrimental effects, smoking is strongly advised against, particularly for individuals who are survivors of colon cancer.

In the context of our research, alcoholic drinking was delineated as a contributory factor to mortality variance among three distinct CCI groups. This factor exacerbates clinical outcomes by engendering extended hospital stays, protracted recovery durations, escalated healthcare expenditures, and an increase in both general and cancer-specific mortality rates^32^, ^33^. Excessive alcohol consumption was found to be strongly linked to an increased risk of early-onset colon cancer (EOCRC), regardless of polygenic risk score (PRS) levels, with its effect on EOCRC risk comparable to having a significantly higher genetically determined risk^34^. The study suggests that avoiding heavy drinking could notably reduce the risk of colon cancer, especially EOCRC, akin to possessing a considerably lower genetic risk for the disease.

Our study also revealed that an increasing overall mortality was with higher CCI scores. This finding indicated that comorbidities significantly affect survival outcomes in colon cancer patients. The analysis of specific causes of death in the CCI, including colon cancer-specific mortality, followed similar trends. For example, diabetes mellitus is significantly associated with an increased risk of colon cancer, as evidenced by a meta-analysis, which found that diabetics have a 38% higher risk of colon cancer (RR: 1.38, 95% CI: 1.26-1.51), even after controlling for smoking, obesity, and physical activity^35^. Additionally, diabetes influences the prognosis of colon cancer. For instance, a cohort study demonstrated that individuals with type 2 diabetes had higher cancer-specific mortality in non-metastatic colon cancer, independent of insulin levels^36^. Despite these findings, the increased risk associated with diabetes is not considered sufficient to modify current colon cancer surveillance recommendations based on age and other factors.

Our study indicated the importance of personalized treatment approaches for cancer patients. Proactive monitoring and management of comorbidities with cancer therapy could improve survival rates and the quality of life for colon cancer survivors with comorbidities^37^. Patients with severe comorbidities may require an integrated and multidisciplinary care model to balance cancer management with the control of coexisting conditions^38^, ^39^. Especially for high-risk patients, personalized treatment plans should include close collaboration between oncologists and other specialists, such as cardiologists or endocrinologists, to address the patient's whole health profile rather than only focusing on cancer therapy^40^. Early and aggressive management of comorbidities should be integrated into the cancer care pathway to reduce their impact on mortality. This is an important clinical implication of this study.

The primary strength of this study lies in providing essential insights into how comorbidities impact the survival of colon cancer patients, emphasizing the necessity for personalized care strategies and the inclusion of comorbidity assessments in treatment planning and prognosis. This research is fundamentally important for healthcare professionals, stressing the need to consider comorbidities for accurate prognostication and effective treatment strategies in colon cancer management. These considerations are critical as they markedly influence patient outcomes, showcasing the diverse risks associated with different comorbidities in this demographic. Moreover, the study's utilization of a longitudinal population database offers a comprehensive perspective on the mortality risk from multiple comorbidities in colon cancer patients.

However, several limitations are present. The retrospective cohort design resulted in a larger proportion of patients diagnosed at early stages (Stages I or II) and a smaller number at Stage IV. In addition, the absence of data on patients' socioeconomic status and lifestyle behaviors, which could sway mortality risk estimates, is a notable gap. Also, the potential for misclassification bias exists due to variations in the definition of comorbidities based on the International Classification of Diseases system's diagnostic codes. This issue, however, has been partly addressed in previous studies^41^. Another limitation is the potential lack of comprehensive patient evaluations, as registry data may not capture the primary cause of death or recurrence details thoroughly, especially when patients discontinue treatments. Nonetheless, the linkage of the TCR to the cause-of-death database and the National Health Insurance research database partially mitigates this issue by enabling extensive patient follow-up. Moreover, our study lacks a detailed analysis of the impact of specific comorbidities, such as diabetes or cardiovascular disease, on mortality risk. Although the CCI provides a composite measure of comorbidity burden, the effects of individual conditions are worth exploring. The mortality risk of specific high-risk comorbidities in colon cancer survivors should be investigated in the future research to offer more targeted insights for clinical management. Additionally, the high proportion of colon cancer survivors in our study with a CCI score of 0, exceeding 80%, may represent a selectively healthier survivor population. Such survivorship bias could impact the generalizability of our findings to colon cancer patients with a broader comorbidity burden. Future research should include cohorts with a more diverse range of comorbidity profiles to validate these results. Finally, due to the constraints of administrative registry databases, important information such as lifestyle, dietary habits, family history of colon cancer, functional status, physical activity, and colon cancer screening—which could be independently associated with mortality risk—was not exhaustively recorded.

## Conclusion

In conclusion, this research provides critical understanding of the impact of comorbid conditions on the survival outcomes among long-term colon cancer survivors. It highlights the imperative for tailored care approaches, particularly for those patients identified as high-risk, and stresses the significance of incorporating comorbidity assessments into the strategic planning of treatment and the evaluation of prognostic outcomes. Furthermore, the study underscores the importance of effectively managing comorbidities, especially in patients with elevated CCI scores, as a key component of comprehensive cancer care.

## Figures and Tables

**Table 1:** The baseline information between colon cancer survivors with no, mild, and severe comorbidities

	Overall (n=13209)	No comorbidity (n=10862)	Mild comorbidity (n=1325)	Severe comorbidity (n=1022)	p-value
Age, mean ± SD	68.84 ± 12.65	67.78 ± 12.63	74.37 ± 11.19	72.90 ± 11.97	<0.0001
Age group					<0.0001
<65	4868(36.85)	4355(40.09)	262(19.77)	251(24.56)	
>=65	8341(63.15)	6507(59.91)	1063(80.23)	771(75.44)	
Sex					<0.0001
Male	7360(55.72)	5932(54.61)	793(59.85)	635(62.13)	
Female	5849(44.28)	4930(45.39)	532(40.15)	387(37.87)	
BMI	24.21 ± 3.91	24.12 ± 3.86	24.63 ± 4.10	24.69 ± 4.08	<0.0001
BMI group					<0.0001
<18.5	706(5.34)	596(5.49)	59(4.45)	51(4.99)	
18.5-24	7456(56.45)	6235(57.40)	700(52.83)	521(50.98)	
>=25	5047(38.21)	4031(37.11)	566(42.72)	450(44.03)	
Smoking					<0.0001
Never	9676(73.25)	8048(74.09)	950(71.70)	678(66.34)	
Ever/current	3533(26.75)	2814(25.91)	375(28.30)	344(33.66)	
Drinking					0.0181
Never	10410(78.81)	8595(79.13)	1045(78.87)	770(75.34)	
Ever/current	2799(21.19)	2267(20.87)	280(21.13)	252(24.66)	
Cancer Stage					0.0055
Early stage	7117(53.88)	5846(53.82)	756(57.06)	515(50.39)	
Late stage	6092(46.12)	5016(46.18)	569(42.94)	507(49.61)	
Treatment					
Surgery	12979(98.26)	10666(98.20)	1309(98.79)	1004(98.24)	0.2920
Radiotherapy	1617(12.24)	1311(12.07)	158(11.92)	148(14.48)	0.0744
Chemotherapy	7348(55.63)	6042(55.63)	720(54.34)	586(57.34)	0.3495
Outcome					
Death	1255(9.50)	499(4.59)	285(21.51)	471(46.09)	<0.0001
Time to death	1.46 ± 0.85	1.77 ± 0.80	1.33 ± 0.82	1.22 ± 0.82	<0.0001

**Table 2:** The risk of overall mortality among colon cancer survivors between three comorbidities burden groups.

	Patients	Death	Crude HR	p-value	Adjusted HR*	p-value
CCI group						
CCI=0	10862	499	Ref		Ref	
CCI=1,2	1325	285	5.26(4.55,6.08)	<0.0001	4.56(3.93,5.28)	<0.0001
CCI>=3	1022	471	13.87(12.22,15.74)	<0.0001	12.67(11.15,14.40)	<0.0001
Age						
<65	4868	154	Ref		Ref	
>=65	8341	1101	4.40(3.72,5.21)	<0.0001	3.39(2.86,4.03)	<0.0001
Sex						
Male	7360	773	Ref		Ref	
Female	5849	482	0.78(0.69,0.87)	<0.0001	0.89(0.78,1.02)	0.0887
BMI						
<18.5	706	98	1.47(1.19,1.81)	0.0004	1.68(1.36,2.08)	<0.0001
18.5-24	7456	719	Ref		Ref	
>=25	5047	438	0.90(0.80,1.01)	0.0685	0.77(0.68,0.87)	<0.0001
Smoking						
Never	9676	859	Ref		Ref	
Ever/current	3533	396	1.28(1.14,1.44)	<0.0001	1.22(1.05,1.41)	0.0085
Drinking						
Never	10410	983	Ref		Ref	
Ever/current	2799	272	1.03(0.90,1.18)	0.6883	0.90(0.77,1.04)	0.1576
Cancer Stage						
Early stage	7117	651	Ref		Ref	
Last stage	6092	604	1.09(0.98,1.22)	0.1310	1.11(0.99,1.25)	0.0733
Treatment						
Surgery (ref: no)	12979	1233	0.99(0.65,1.52)	0.9776	0.84(0.55,1.29)	0.4273
Radiotherapy (ref: no)	1617	132	0.83(0.70,0.99)	0.0455	0.85(0.70,1.03)	0.0935
Chemotherapy (ref: no)	7348	672	0.91(0.82,1.02)	0.1074	0.96(0.85,1.08)	0.4610

*Adjusted Age, Sex, BMI, Smoking, Drinking, Cancer stage, Surgery, Radiotherapy, Chemotherapy

## Abbreviations

CCI: Charlson Comorbidity Index
SD: standard deviation
HR: hazard ratio
CI: confidence interval
TCR: Taiwan Cancer Registry
HWDC: Health, and Welfare Data Science Center
BMI: Body Mass Index
IRB: Institutional Review Board
CMMC: Chi Mei Medical Center
AHRs: Adjusted Hazard Ratios
HNPCC: hereditary nonpolyposis colon cancer
EOCRC: early-onset colon cancer
PRS: polygenic risk score
